# Trends of Acute Myocardial Infarction Mortality in People Over 65 Years Old in the United States From 1999–2020: Insight From the CDC-WONDER Database

**DOI:** 10.7759/cureus.58225

**Published:** 2024-04-14

**Authors:** Bekure B Siraw, Didien Meyahnwi, Yordanos T Tafesse, Biruk B Siraw, Juveriya Yasmeen, Samrawit Melka

**Affiliations:** 1 Internal Medicine, Ascension Saint Joseph Hospital, Chicago, USA; 2 Public Health, University of California Berkeley, Berkeley, USA; 3 Biological Sciences, The University of Chicago Medicine, Chicago, USA; 4 Medical Biotechnology, University of Eastern Piedmont, Novara, ITA

**Keywords:** united states, elderly, disparities, mortality trends, acute myocardial infarction

## Abstract

Background

Over the past two decades, there have been numerous advances in acute myocardial infarction (AMI) care. We assessed the impact of these advances on the trend of AMI-related mortality.

Methods

This retrospective analysis of the Centers for Disease Control’s Wide-ranging Online Data for Epidemiologic Research (CDC_WONDER) database focused on AMI-related mortality in individuals aged 65 and older in the United States from 1999 to 2020. Trends -n crude and age-adjusted mortality rates (AAMR) were assessed based on socio-demographic and regional variables using Joinpoint Regression software (Joinpoint Regression Program, Version 5.0.2 - May 2023; Statistical Methodology and Applications Branch, Surveillance Research Program, National Cancer Institute Bethesda, Maryland). Annual percentage change (APC) with 95% confidence intervals (CIs) for the AAMRs were calculated for the line segments linking a Joinpoint using a data-driven weighted Bayesian Information Criterion (BIC) model.

Results

There were 2,354,971 AMI-related deaths with an overall decline in the AAMR from 474.6 in 1999 to 153.2 in 2020 and an average annual percentage change (AAPC) of -5.3 (95% CI -5.4 to -5.2). Notable declines were observed across gender, race, age groups, and urbanization levels. However, the rate of AMI-related deaths at decedents’ homes slowed down between 2008 and 2020 and climbed up between 2018 and 2020. In addition to this, nonmetropolitan areas were found to have a significantly lower decline in mortality when compared to large and medium/small metropolitan areas.

Conclusion

While there is an overall positive trend in reducing AMI-associated mortality, disparities persist, emphasizing the need for targeted interventions.

## Introduction

Heart disease has remained the leading cause of mortality in the United States for more than six decades [1]. According to a 2014 report from the AHA, approximately every 34 seconds, one American has a coronary event, and approximately every minute and 23 seconds, an American will die of one [2]. Age is a powerful predictor of adverse events after an acute coronary event [3-5]. With the lengthening of life expectancy, it is projected that from 2000 to 2030, the proportion of people ≥65 years of age will increase from 12.4% to 19.6% in the United States [6]. Acute myocardial infarction (AMI) remains a critical cardiovascular event with substantial implications for public health, particularly among individuals aged 65 and older in the United States. [7] Over the past two decades, there have been numerous advances in AMI care, including prompt and increasing use of early reperfusion with primary percutaneous coronary intervention (PCI), development of newer and potent antiplatelet therapy, and designing systems of care management, that have resulted in improvement of overall AMI mortality [5,8-12]. Despite advancements in medical care and a declining overall cardiovascular mortality rate, a more nuanced examination of AMI-associated mortality is imperative. This study aims to build upon existing knowledge by leveraging the Centers for Disease Control and Prevention - Wide-ranging Online Data for Epidemiologic Research (CDC-WONDER) database, a comprehensive repository of mortality data, to elucidate temporal trends in AMI-associated deaths among individuals aged 65 and older. Insights from this analysis are poised to inform targeted interventions, thereby contributing to the overall cardiovascular health of the aging population.

## Materials and methods

This retrospective study utilized data from the CDC-WONDER's multiple cause of death database. This publicly accessible repository compiles mortality data of US residents derived from death certificates throughout the United States. Each death certificate contains a single underlying cause of death, up to twenty additional multiple causes, and demographic data [13]. The study focused on individuals aged 65 and older who experienced mortality between 1999 and 2020, with acute myocardial infarction documented as the underlying cause of death on their death certificates. AMI-related mortality was identified using the International Classification of Diseases, 10th Revision, Clinical Modification codes I21.0, I21.1, I21.2, I21.3, I21.9, I22.0, I22.1, I22.8, and I22.9 [14]. The study was exempt from institutional review board approval because of the CDC WONDER database containing anonymized, publicly available data.

Data on demographic and regional groups were extracted including age, gender, race/ethnicity, location (region, state, and county), and place of death. Age was recorded as a categorical variable with 10-year increments; 65-74 years, 75-84 years, and >85 years. AMI-related crude and age-adjusted mortality rates were calculated per 100,000 population. For urban-rural classifications, the National Center for Health Statistics Urban-Rural Classification Scheme was used to divide the population into large metropolitan areas (population ≥1 million), medium/small metropolitan areas (population 50,000 to 999,999), and non-metropolitan (population <50,000) counties per the 2013 United States census classification [15]. Regions were classified into Northeast, Midwest, South, and West according to the Census Bureau definitions [16]. Location of death included medical facilities (outpatient, emergency room, inpatient, or death on arrival), home, and nursing home/long-term care. Crude mortality rates were calculated by dividing the number of AMI-related deaths by the corresponding United States population. Age-adjusted mortality rates were standardized using the 2000 United States standard population as previously described [17].

To comprehend the nuanced nature of AMI-associated mortality trends, we analyzed variations in mortality rates over time across categories such as age, gender, race/ethnicity, place of death, and location (region, state, and county). Temporal patterns were visually represented through line and dot graphs and maps. The Joinpoint Regression Program (Version 5.0.2 - May 2023; Statistical Methodology and Applications Branch, Surveillance Research Program, National Cancer Institute Bethesda, Maryland) was used to determine trends in mortality within the study period [18]. This program identifies significant changes in annual mortality trends over time through Joinpoint regression, which fits models of linear segments where significant temporal variation occurred. Annual percentage change (APC) with 95% confidence intervals (CIs) for the AAMRs were calculated for the line segments linking a Joinpoint using a data-driven weighted Bayesian Information Criterion (BIC) model [19]. The weighted averages of the APCs were calculated and reported as average annual percentage changes (AAPC) with corresponding 95% CIs for the reported mortality trend for the entire study period. APC and AAPCs were considered increasing or decreasing if the slope describing the change in mortality over the time interval was significantly different from zero using a 2-tailed t-test. Statistical significance was set at p ≤0.05. All data cleaning and visualizations were performed using R statistical software version 4.1.3 (2023, R Foundation for Statistical Computing, Vienna, Austria) [20].

## Results

Between 1999 and 2020, a total of 2,354,971 mortalities were attributed to acute myocardial infarction (AMI). Females accounted for 50.2% of these cases, with 83.6% being White, 9.1% Black, and 4.9% Hispanic individuals. Mortalities were distributed among the age groups of 65-74 years (25.1%), 75-84 years (36.2%), and >85 years (38.7%). Regarding the location of death, 39.4% occurred at an inpatient medical facility, while 24.1%, 18.3%, and 16.9% took place in the decedent’s home, nursing home/long-term care, and emergency room, respectively (Table 1).

**Table 1:** Sociodemographic and regional frequency distribution of acute myocardial infarction-associated mortality rate in people older than 65 years in the United States from 1999 to 2020. CMR - crude mortality rate, AAMR - age-adjusted mortality rate, ER – emergency room * Per 100,000 population.

Variable		Number of Death	CMR	AAMR
Race	Black	215,662	259	278.5
	White	1,967,827	259.6	258.1
	Hispanic	117,016	179.7	198.8
	Asian/Pacific Islander	45639	127.1	141.8
	American Indian/Alaska Native	8827	145.9	166.1
Age	65-74 years	592,729	116.1	-
	75-84 years	851,396	285.2	-
	>85 years	910,846	762.1	-
Gender	Male	1,172,942	292.6	318.9
	Female	1,182,029	224	209.4
Place of Death	Medical Facility - Inpatient	904,575	-	-
	Decedent's home	544,065	-	-
	Nursing home/long-term care	406,455	-	-
	Medical Facility - Outpatient or ER	374,341	-	-
	Medical Facility - Dead on Arrival	31,792	-	-
Region	Northeast	469,041	261.0	251.6
	Midwest	575,479	277.9	273.3
	South	907,320	264.2	273.6
	West	403,131	203.4	207.4
Urbanization	Large Metro	1,084,899	231.4	232.1
	Medium/Small Metro	704,716	246.7	249
	Non-metro	565,356	339.1	343

**Figure 1:**
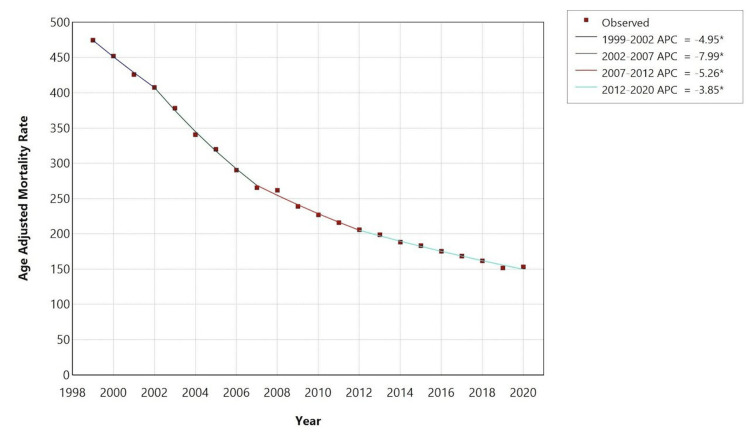
Trend of acute myocardial infarction age-adjusted mortality rate in people older than 65 years in the United States from 1999 – 2020. * indicated p < 0.05; APC – annual percent change.

The overall mean AAMR was 255.4 per 100,000 population. It exhibited a substantial decline from 474.6 in 1999 to 153.2 in 2020, with an AAPC of -5.3 (95% CI -5.4 to -5.2). The APC in AAMR was −4.9 (95% CI −6 to −3.4) from 1999 to 2002, which then accelerated to −8 (95% CI −9.2 to −7.4) from 2002 to 2007 and subsequently decelerated to −5.3 (95% CI −6.8 to −4.3) from 2007 to 2012 and further decelerated to −3.8 (95% CI −4.2 to −2.6) from 2012 to 2020 (Figure 1, Table 2).

**Table 2:** Trend of acute myocardial infarction associated mortality rate in people older than 65 years in the United States from 1999 – 2020 by sociodemographic and regional variables. APC – annual percentage change, AAPC – average annual percentage change, CI – confidence interval, * - significant at p < 0.05.

Variable	Trend 1			Trend 2			Trend 3			Trend 4				Overall (1999-2020)	
	APC, %	95% CI	Years	APC, %	95% CI	Years	APC, %	95% CI	Years	APC, %	95% CI	Years		AAPC, %	95% CI
Age															
65-74 years	-7.7*	-8.7, -7.2	1999-2007	-4.8*	-6.8, -3.1	2007-2012	-2.17*	-2.7, -0.7	2012-2020	N/A	N/A	N/A		-4.9*	-5.1, -4.8
75-84 years	-5.8*	-6.7, -4.3	1999-2002	-8.01*	-9.2, -7.5	2002-2007	-5.2*	-6.8, -4.3	2007-2012	-3.9*	-4.2, -2.5	2007-2012		-5.5*	-5.6, -5.3
>85 years	-2.8*	-4.3, -0.4	1999-2002	-7.9*	-9.6, -7.2	2002-2007	-5.3*	-5.5, -5	2007-2020	N/A	N/A	N/A		-5.6*	-5.7, -5.4
Gender															
Female	-5*	-6.5, -2.9	1999-2002	-7.6*	-8.9, -7.1	2002-2009	-4.7*	-4.9, -4.3	2009-2020	N/A	N/A	N/A		-5.7*	-5.9, -5.6
Male	-5.3*	-6.4, -3.6	1999-2002	-8.1*	-9.3, -7.5	2002-2007	-4.9*	-6.2, -4.1	2007-2012	-3.5*	-3.8, -2.8	2012-2020		-5.2*	-5.3, -5.1
Race/Ethnicity															
Non-Hispanic White	-5.1*	-6.2, -3.1	1999-2002	-7.9*	-9.2, -7.3	2002-2007	-5.3*	-7, -4	2007-2011	-3.9*	-4.3, -2.8	2011-2020		-5.3*	-5.4, -5.1
Non-Hispanic Black	-3.8	-5.7, 0.01	1999-2002	-7.5*	-9.7, -6.9	2002-2009	-4.9*	-5.9, -4.2	2009-2018	0.7	-3.4, 2.7	2018-2020		-5.1*	-5.4, -4.8
Hispanic/Latino	-2.8	-5.4, 1.4	1999-2002	-7.6*	-9.7, -7	2002-2011	-3.7*	-7, -2.7	2011-2018	-1.6	-2.7, 4.7	2018-2020		-4.8*	
Asian/Pacific Islander	-6.2*	-6.8, -5.8	1999-2014	-2.1	-3.7, 1.5	2014-2020	N/A	N/A	N/A	N/A	N/A	N/A		-5.1*	-5.1, -4.5
American Indian/Alaska Native	-6.8*	-12, -5.2	1999-2008	-4.8*	-5.9, -0.1	2008-2020	N/A	N/A	N/A	N/A	N/A	N/A		-5.7*	-6.2, -5.1
Census Region															
Northeast	-4.5*	-6.1, -1.9	1999-2002	-8.4*	-10.3, -7.5	2002-2007	-5*	-5.2, -4.7	2007-2020	N/A	N/A	N/A		-5.8*	-5.9, -5.6
Midwest	-6.9*	-7.4, -6.5	1999-2009	-3.8*	-4.1, -3.4	2009-2020	N/A	NA	N/A	N/A	N/A	N/A		-5.3*	-5.4, -5.1
South	-4.7*	-6, -2.5	1999-2002	-8*	-9.4, -7.3	2002-2007	-5.5*	-6.7, -4.2	2007-2012	-3.7*	-4.1, -2.5	2012-2020		-5.3*	-5.4, -5.1
West	-6.6*	-7.2, -6.2	1999-2011	-3.4*	-4.1, -2.4	2011-2020	N/A	NA	N/A	N/A	N/A	N/A		-5.3*	-5.5, -5
Urbanization															
Large Metro	-4.5	-7.5, -1.4	1999-2002	-7.7	-8.5, -7	2002-2010	-5.2	-5.7, -4.7	2010-2020	N/A	N/A	N/A		-6.1*	-6.6, -5.5
Medium/Small Metro	-4.8	-6.9, -2.5	1999-2002	-7.3	-8, -6.5	2002-2009	-4.3	-4.7, -4.1	2009-2020	N/A	N/A	N/A		-5.4*	-5.8, -5
Non-metro	-6	-6.5, -5.6	1999-2009	-3.4	-3.8, -3	2009-2020	N/A	N/A	N/A	N/A	N/A	N/A		-4.7*	-4.9, -4.4
Place of death															
Medical Facility - Inpatient	-6.6*	-7.4, -6	1999-2010	-2.8*	-3.5, -1.8	2010-2020	N/A	N/A	N/A	N/A	N/A	N/A		-4.8*	-5.1, -4.5
Decedent's home	-3.5*	-4.5, -2.9	1999-2009	0.9	-3.1, 1.7	2009-2018	6.5*	1.5, 9.1	2018-2020	N/A	N/A	N/A		-0.7*	-1.1, -0.4
Nursing home/long-term care	-1.9	-4, 1.56	1999-2002	-6.4*	-8.7, -5.7	2002-2009	-4.1*	-4.4, -3.3	2009-2020	N/A	N/A	N/A		-4.5*	-4.7, -4.31
Medical Facility - Outpatient or ER	-6*	-6.4, -5.6	1999-2008	0.5	-0.6, 2.1	2008-2012	-2.2*	-2.8, -1.9	2012-2020	N/A	N/A	N/A		-3.4*	-3.5, -3.2
Medical Facility - Dead on Arrival	-13.5*	-18.1, -11	1999-2006	-7.6*	-8.4, -6.4	2006-2020	N/A	N/A	N/A	N/A	N/A	N/A		-9.6*	-10.1, -9
Overall	-4.9*	-5.9, -3.4	1999-2002	-8*	-9.2, -7.4	2002-2007	-5.3*	-6.8, -4.3	2007-2012	-3.8*	-4.2, -2.6	2012-2020		-5.3*	-5.5, -5.2

**Figure 2:**
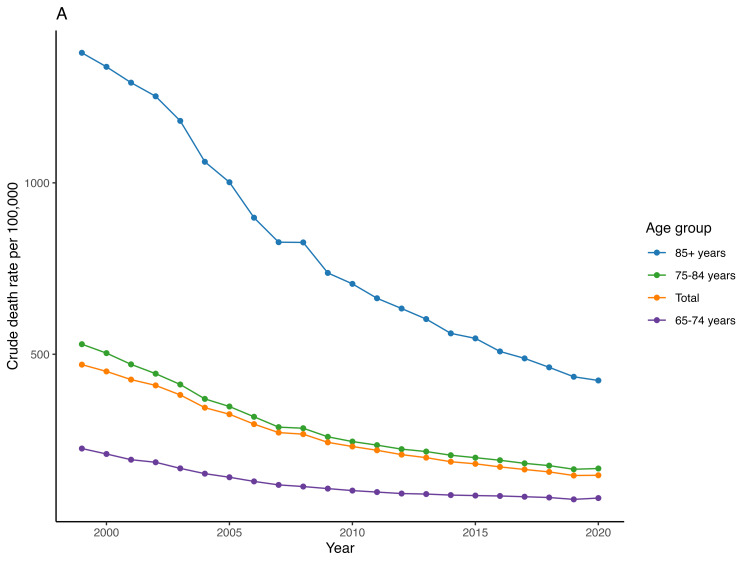
Trend of acute myocardial infarction-associated crude mortality rate in people older than 65 years in the United States from 1999 – 2020 across different age groups.

People older than 85 years had the highest mean CMR (762.1), followed by people 75 to 84 years old 285.2) and people 65 to 74 years old (198.8). The mean CMR in people older than 85 years old declined from 1379.3 in 1999 to 423.5 in 2020 [AAPC = -5.6 (95% CI -5.7 to -5.4)] while in people 75 to 84 years old it decreased from 529.3 in 1999 to 166.7 in 2020 (AAPC = -5.5 (95% CI -5.6 to -5.3)) and in people 65 to 74 years old it decreased from 225 in 1999 to 80.5 in 2020 [AAPC of -4.9 (95% CI -5.1 to -4.8)] (Figure 2, Table 2). We found no significant difference in the APC between different age groups (all p values > 0.05).

**Figure 3:**
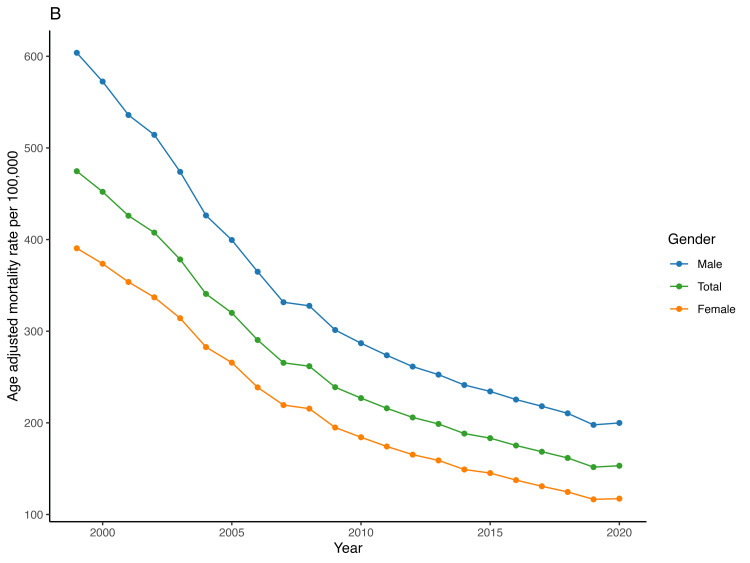
Trend of acute myocardial infarction-associated age-adjusted mortality rate in people older than 65 years in the United States from 1999 – 2020 across different genders.

Men (318.9) exhibited a higher mean age-adjusted mortality rate compared to women (209.4). The mean AAMR in women declined from 390.5 in 1999 to 117.3 in 2020, with an AAPC of -5.7 (95% CI -5.9 to -5.6) while in men it decreased from 603.8 in 1999 to 199.9 in 2020, with an AAPC of -5.2 (95% CI -5.3 to -5.1) (Figure 2, Table 2). We found no significant difference in the APC between men and women [AAPC difference -0.5 (95% CI -1.2 to 0.1)] (Figure 3, Table 2).

**Figure 4:**
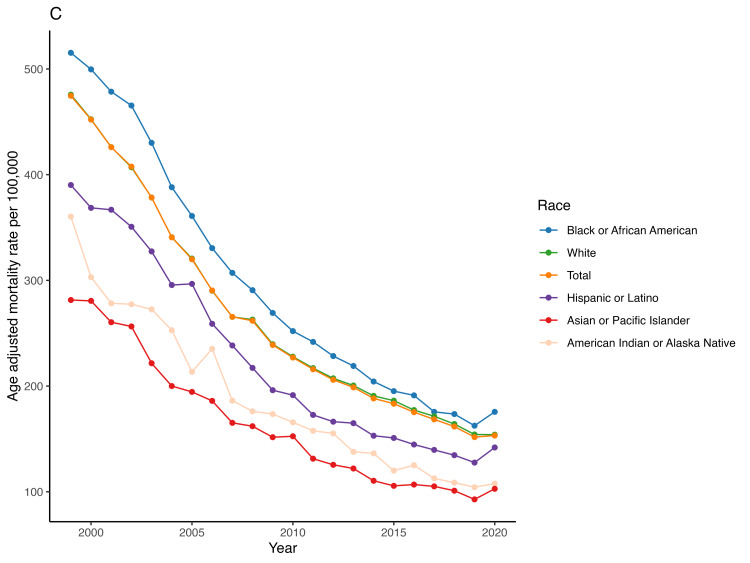
Trend of acute myocardial infarction-associated age-adjusted mortality rate in people older than 65 years in the United States from 1999 – 2020 across different racial/ethnic groups.

The mean AAMR was highest in Blacks (278.5), followed by Whites (258.1) and Hispanics (198.8). The mean AAMR in Blacks declined from 515.3 in 1999 to 175.6 in 2020, with an AAPC of -5.1 (95% CI -5.4 to -4.8) while in Whites it decreased from 475.7 in 1999 to 154 in 2020, with AAPC of -5.3 (95% CI -5.4 to -5.1) and in Hispanics it decreased from 390.2 in 1999 to 141.9 in 2020, with an AAPC of -4.8 (95% CI -5.1 to -4.5) (Figure 4, Table 2). We found no significant difference in the APC between different racial groups (all p values > 0.05) (Figure 4, Table 2).

**Figure 5:**
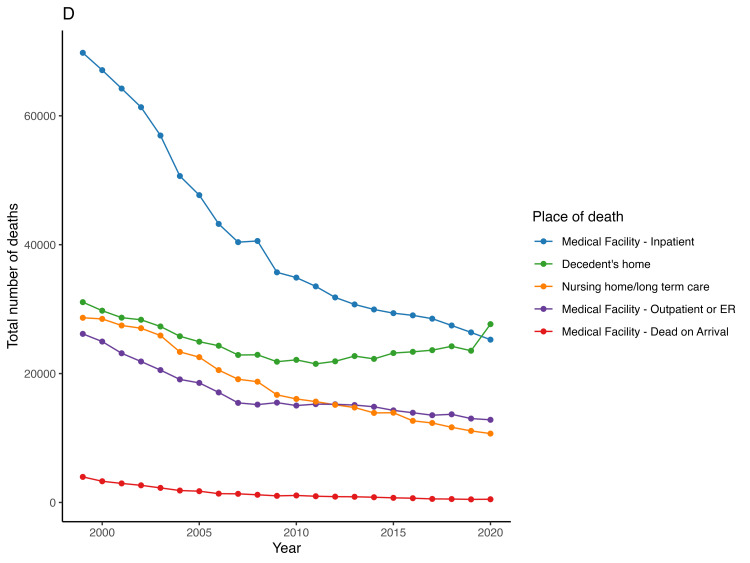
Trend of acute myocardial infarction-associated number of deaths in people older than 65 years in the United States from 1999 – 2020 by place of death.

The APC for the number of AMI mortalities that occurred at the decedents’ household dropped from -3.5 (95% CI -4.5 to -2.9) from 1999 to 2009 and then remained steady from 2009 to 2018 [APC = 0.9 (95% CI -3.1 to 1.7)] but it has been climbing up from 2018 to 2020 [APC = 6.5 (95% CI 1.5 to 9)]. Deaths occurring on arrival at a medical facility have drastically declined with an APC of -13.5 (95% CI -18.2.1 to -11.4) from 1999 to 2006 and -7.6 (95% CI -8.4 to -6.4) from 2006 to 2020. Deaths occurring in inpatient medical facilities, outpatient settings or emergency rooms, nursing homes, and long-term care settings have also been steadily declining during the study period (Figure 5, Table 2). 

**Figure 6:**
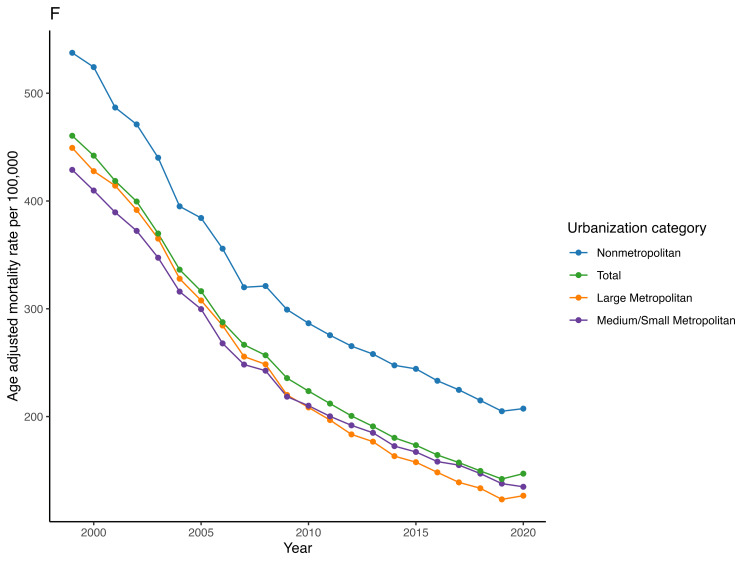
Trend of acute myocardial infarction-associated age-adjusted mortality rate in people older than 65 years in the United States from 1999 – 2020 across different urbanization levels.

The mean AAMR in the large metropolitan areas was 232.1 whereas, in medium/small metropolitan areas and nonmetropolitan areas, it was 249 and 343 respectively. The mean CMR in large metropolitan areas declined from 449.3 in 1999 to 126.6 in 2020 [AAPC = -6.1 (95% CI -6.6 to -5.6)] while in medium/small metropolitan areas it decreased from 428.8 in 1999 to 134.8 in 2020 [AAPC = -5.4 (95% CI -5.8 to -5)] and in nonmetropolitan areas it decreased from 537.5 in 1999 to 207.3 in 2020 [AAPC of -4.7 (95% CI -4.9 to -4.4)] (Figure 6, Table 2). Nonmetropolitan areas were found to have a significantly lower AAPC when compared to large metropolitan (AAPC difference -1.4 (95% CI -2 to -0.8) and medium/small metropolitan [AAPC difference -0.7 (95% CI -1.2 to -0.3)] areas.

**Figure 7:**
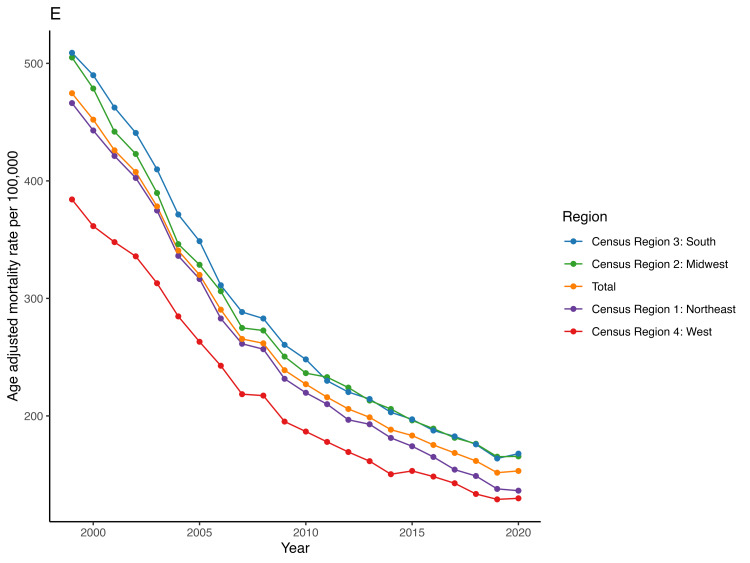
Trend of acute myocardial infarction-associated age-adjusted mortality rate in people older than 65 years in the United States from 1999 – 2020 across different census regions.

At a regional level, the South (273.3) and Midwest (273.6) regions had the highest mean AAMR whereas the West (207.4) region had the lowest. In the Northeast region, AAMR declined from 466.2 in 1999 to 136.4 in 2020 (AAPC = -5.8 (95% CI -5.9 to -5.6)) while in the West, it fell from 384.2 in 1999 to 129.9 in 2020 [AAPC = -5.3 (95% CI -5.5 to -5)]. Similarly, it decreased from 505 in 1999 to 165.6 in 2020 [AAPC = -5.3 (95% CI -5.4 to -5.1)] in the Midwest, and in the South, it dropped from 509 in 1999 to 167.9 in 2020 [AAPC = -5.3 (95% CI -5.4 to -5.1)] (Table 2, Figure 7). We found no significant difference in the APC between different regions (all p values > 0.05).

**Figure 8:**
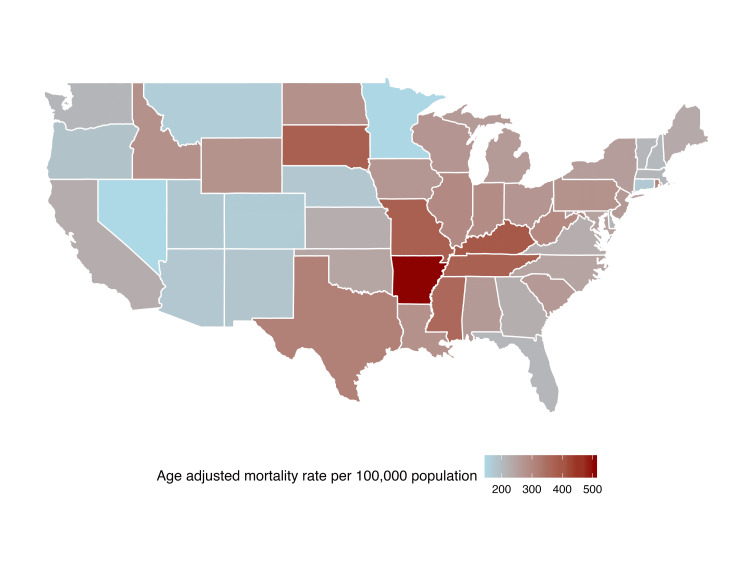
Myocardial infarction associated mean age-adjusted mortality rate by state in people older than 65 years in the United States from 1999 – 2020.

Among the states, Arkansas (513.8), Kentucky (389.2), and South Dakota (376.9) recorded the highest mean AAMR, whereas Alaska (129.8), Nevada (144.3), and Minnesota (147.0) reported the lowest rates (Figure 7). At a county level, Desha County in Arkansas (1366.0), Perry County in Kentucky (1147.3), and Greene County in Mississippi (1147.3) exhibited the highest mean AAMR, while Pitkin County in Colorado (38.6), Eagle County in Colorado (55.0), and Storey County in Nevada (55.8) demonstrated the lowest AAMR. (Figure 8).

## Discussion

Our study provides valuable insights into the 20-year trends of AMI-associated mortality among individuals aged 65 and older in the United States. Most impressively, there has been a substantial overall decline in age-adjusted AMI mortality rate from 1999 to 2020. This declining trend is seen across all pre-specified age groups ≥ 65, races, genders, geographic/Census regions, and urbanization levels. Nevertheless, the decline in AMI-related mortalities at decedents' homes halted after 2008 and saw a sharp increase in 2020.

The general decrease in AMI-associated mortality is likely due to broader advancements in AMI care over the past two decades, including the increased use of early revascularization with primary percutaneous coronary intervention (PCI), advancements in antiplatelet therapy, and improved systems of care management [8-12,21].

While there was a consistent decrease in AMI-related deaths that occurred at home from 1999 to 2009, the rates have held steady between 2009 and 2018 and have trended slightly upwards between 2018 and 2020. The financial recession that hit the US in 2008, leading to mass layoffs and loss of medical insurance could have prompted healthcare underutilization, delayed presentation to the hospital in advanced stages of disease, and aberrant social practices like alcohol overuse and drug use which could have been triggers for acute coronary events [22]. Another potential explanation for this trend of AMI-related deaths at home is the increase in the utilization of home hospice services for eligible patients who would eventually have acute coronary events as the immediate cause of their mortality [23]. The abrupt jump in the home AAMR for myocardial infarction (MI) in 2020 could be the result of the COVID-19 pandemic which placed considerable pressure on the healthcare system, led to fears of healthcare utilization prompting people to attempt home remedies for medical emergencies, and presented late to the hospital. This trend reversal in the first year of the pandemic was more pronounced in Black and Hispanic populations, groups that were disproportionately affected by the pandemic [24].

Race and ethnicity relative differences in the AAMR were consistent with what has been previously described with Blacks exhibiting the highest mean age-adjusted mortality rate, followed by Whites and Hispanics [22]. These outcome disparities are mostly driven by structural racism and the social determinants of health [25]. Though pairwise comparisons did not reveal any significant differences in the rate of mortality decrease between the various race/ethnicity groups, the persistently higher rates of AMI-related deaths among blacks throughout the study period highlight differences in healthcare outcomes by race and reinforce the need for targeted interventions to bridge these outcome gaps.

Though CMR decreased significantly in all age groups, individuals aged >85 years notably exhibited the highest AMI-related CMR throughout the study years. This finding is consistent with existing literature and emphasizes the vulnerability of this age group to AMI-related mortality. The increased vulnerability is due to a variety of factors including a higher burden of comorbidities, increased time between symptom onset and presentation to the hospital, and atypical presentation of AMI which can lead to delayed diagnosis and treatment [26]. The above-mentioned factors further decrease the likelihood of most elderly patients receiving evidence-based, mortality-decreasing interventions [27].

Geographical differences in AMI-associated mortality rates were identified at both regional and County levels. While all Census regions saw significant declines in mortality, the South and Midwest regions consistently experienced the highest age-adjusted mortality rates. These regions encompass most of the Southern and rural “Stroke belt” states that historically have had the highest cardiovascular disease (CVD) mortality [28]. It is well documented that regions and counties with the greatest CVD mortality have a preponderance of factors that lead to high AMI incidence and poor outcomes such as high prevalence of underinsured/uninsured adults, smoking, and obesity [28]. This suggests a complex interplay of regional variations of demographic, socio-economic, and healthcare system factors and helps explain the heterogeneity in AMI mortality across US geographical divisions. 

Although the AMI-related AAMR decreased in both rural and urban areas in the US, pairwise comparisons revealed large and medium-sized metros independently had significantly higher rates of decline than Nonmetro (rural areas). This is similar to previously reported findings and could be explained by conditions in rural areas that put residents at higher risk of death including long travel distances to specialty and emergency care services, higher rates of cigarette smoking, obesity, high blood pressure, and paucity of grocery stores that provide affordable and healthy foods [28]. AMI patients who presented in rural hospitals were also less likely to receive important, aggressive, evidence-based procedures like diagnostic catheterizations, percutaneous interventions, and coronary artery bypass grafts, which have been proven to reduce AMI-related mortality [29].

While this study contributes valuable insights, it is essential to acknowledge its limitations. The data, derived from death certificates, may have inherent reporting biases or inaccuracies in cause-of-death attribution. Additionally, the study does not explore individual-level risk factors or variations in treatment modalities, limiting the depth of the analysis. Future research could benefit from incorporating more granular data and considering additional factors that might influence AMI-related mortality. Despite these limitations, this study is based on the CDC-WONDER database, which is a comprehensive repository of mortality data in the US, a statistic that is reported with excellent accuracy. The study spans a fairly extensive period (two decades) and cements existing knowledge on the evolution of AMI-related mortality while demonstrating areas that need specific public health and policy interventions. 

## Conclusions

Our study provides a comprehensive analysis of the trends in AMI-associated mortality among individuals aged 65 and older in the US from 1999 to 2020. The observed decline in mortality rates reflects the positive impact of advancements in AMI care. Though there has been a general decline in AMI-related mortality, disparities persist between race groups, geographical regions, and urbanization levels. The COVID-19 pandemic also seems to have clawed back the progress made in reducing AMI-related deaths over the years. With an increasingly aging US population, governments and policymakers need to implement targeted social, economic, and health policy decisions to help bridge these gaps in outcomes to maintain or accentuate the decline in cardiovascular disease mortality.
